# Age-Predicted Maximal Heart Rate in Recreational Marathon Runners: A Cross-Sectional Study on Fox's and Tanaka's Equations

**DOI:** 10.3389/fphys.2018.00226

**Published:** 2018-03-15

**Authors:** Pantelis T. Nikolaidis, Thomas Rosemann, Beat Knechtle

**Affiliations:** ^1^Exercise Physiology Laboratory, Nikaia, Greece; ^2^Institute of Primary Care, University of Zurich, Zurich, Switzerland; ^3^Medbase St. Gallen Am Vadianplatz, St. Gallen, Switzerland

**Keywords:** age groups, cardiac rate, endurance runners, graded exercise test, sex

## Abstract

Age-based prediction equations of maximal heart rate (HR_max_), such as the popular formulas Fox's 220-age, or Tanaka's 208-0.7 × age, have been widely used in various populations. Surprisingly, so far these equations have not been validated in marathon runners, despite the importance of the role of HR_max_ for training purposes in endurance running. The aim of the present study was to examine the validity of Fox and Tanaka equations in a large sample of women and men recreational marathon runners. Participants (*n* = 180, age 43.2 ± 8.5 years, VO_2max_ 46.8 mL/min/kg, finishers in at least one marathon during the last year) performed a graded exercise test on a treadmill, where HR_max_ was measured. Measured HR_max_ correlated largely with age in the total sample (*r* = −0.50, *p* < 0.001), women (*r* = −0.60, *p* < 0.001) and men (*r* = −0.53, *p* < 0.001). In women, a large main effect of method on HR_max_ (*p* = 0.001, η^2^ = 0.294) was shown with measured HR_max_ lower than Fox-HR_max_ (−4.8 bpm; −8.4, −1.3) and Tanaka-HR_max_ (−4.9 bpm; −8.1, −1.8). In men, a moderate effect of assessment method on HR_max_ was found (*p* = 0.001, η^2^ = 0.066) with measured HR_max_ higher than Fox-HR_max_ (+2.8; 1.0, 4.6), Tanaka-HR_max_ higher than Fox-HR_max_ (+1.2; 0.7, 1.7). Based on these findings, it was concluded that Fox and Tanaka' formulas overestimated HR_max_ by ~5 bpm in women, whereas Fox underestimated HR_max_ in men by ~3 bpm. Thus, we recommend the further use of Tanaka's formula in men marathon runners. In addition, exercise physiologists and sport scientists should consider the observed differences among various assessment methods when performing exercise testing or prescribing training program relying on HR.

## Introduction

Training intensity and volume are predictors of performance in marathon runners (Schmid et al., 2012). A daily task in the context of training is to run at an optimal intensity in order to elicit the desired physiological adaptations, such as increased speed at anaerobic threshold and maximal oxygen uptake (Lepers and Stapley, 2016). If the intensity is inadequate, the stimulus for these adaptations is missing. On the other hand, if the intensity exceeds the optimal level, the risk of overtraining increases (O'Connor, 2007). Thus, it is important to assess exercise intensity accurately, which relies on objective measures such as heart rate (HR), oxygen uptake and lactate, and subjective methods such as rate of perceived exertion (Foster et al., 2017). When HR is used as a measure of intensity, usually it is expressed as a function of maximal HR (HR_max_) (Vesterinen et al., 2017).

HR_max_ can be measured using a graded exercise test (GXT) either in a laboratory or in the field (Cleary et al., 2011; Nikolaidis, 2015). However, occasionally it is not desirable to perform a GXT (e.g., to avoid the fatigue induced by maximal exercise testing close to a race or the associated financial cost). In such case, an alternative is to predict HR_max_ from an age-based equation, considering the inversely proportional relationship between age and HR_max_. The most widely used formulas are those of Fox, Naughton, and Haskell (Fox-HR_max_ = 220 − age) (Fox et al., 1971) and of Tanaka, Monahan, and Seals (Tanaka-HR_max_ = 208 − 0.7 × age) (Tanaka et al., 2001). These equations have been examined extensively in specific categories of adult population such as healthy (Nes et al., 2012), sedentary (Sarzynski et al., 2013), overweight (Franckowiak et al., 2011) and athletes (Faff et al., 2007).

Whereas the abovementioned studies have addressed many issues with regards to the validity of these popular equations of HR_max_, there are some aspects that need further research. For instance, endurance athletes (e.g., marathon runners) and especially master athletes are under-represented in this body of research. A comparison of athletes and non-athletes had revealed lower measured-HR_max_ in the former group (Lester et al., 1968). In a recent study, it was shown that athletes of speed/power sports had similar measured-HR_max_ with endurance athletes and both had lower values than those who were untrained (Kusy and Zielinski, 2012). The decrease in HR_max_ induced by endurance training might be explained by accompanying plasma volume expansion, enhanced baroreflex function, alteration of the electrophysiology of the sinoatrial node and decreased beta-adrenergic receptor number and density (Zavorsky, 2000). Since their measured HR_max_ differs, it is reasonable to assume that the same equation of HR_max_ cannot fit in both athletes and non-athletes. This difference between athletes and non-athletes highlights the need to further examine the popular prediction equations in more samples of athletes. Considering the increasing number of those participating in marathon races (Jokl et al., 2004), the knowledge of the validity of the popular age-based equations has practical application for a large number of recreational marathon runners. Moreover, the age-based prediction of HR_max_ is a major interest for exercise physiologists when administering a GXT, where the achievement of a particular percentage of predicted HR_max_ might be necessary in order to consider the end values as maximal (Schaun, 2017). Therefore, the aim of the present study was to examine the validity of Fox-HR_max_ and Tanaka-HR_max_ in a large sample of recreational marathon runners. The research hypothesis was that these equations, which had been developed in non-athletes, would overestimate HR_max_ in recreational marathon runners due to their expected lower HR_max_ compared to non-athletes (Lester et al., 1968; Zavorsky, 2000; Kusy and Zielinski, 2012).

## Materials and methods

### Study design and participants

One hundred eighty-five recreational marathon runners mostly from the area of Athens volunteered to participate in this study, which had been advertised through popular websites for endurance runners. During September and October 2017, the participants visited the laboratory where they performed a GXT on a treadmill. This study was carried out in accordance with the recommendations of the Institutional Review Board of Exercise Physiology Laboratory Nikaia with written informed consent from all participants. All participants gave written informed consent in accordance with the Declaration of Helsinki. The protocol was approved by the Institutional Review Board of Exercise Physiology Laboratory Nikaia. One participant withdrew from the study during the GXT, whereas four participants did not achieve the criteria of VO_2max_ achievement, and consequently their data excluded from further analysis. Therefore, we included 180 participants from the initial sample. With regards to their sport experience, the median number of marathon completed in the past was 3 and the interquartile range was 2–6. Personal record was 4:09 ± 0:45 h:min.

### Protocols and equipment

#### Anthropometry

Height, body mass, and skinfolds were measured with participants in minimal clothing and barefoot. An electronic weighing scale (HD-351; Tanita, Arlington Heights, IL, USA) was employed for measurement of body mass (to the nearest 0.1 kg), a portable stadiometer (SECA Leicester, UK) for height (0.001 m), and a caliper (Harpenden, West Sussex, UK) for skinfolds (0.2 mm). Body mass index was calculated as the quotient of body mass (kg) to height squared (m^2^), and body fat (BF) was estimated from skinfolds (Parizkova, 1978).

#### Graded exercise test

A modified version of Conconi test was used to assess VO_2max_ (Conconi et al., 1982). Briefly, after a 20-min warm-up including jogging and stretching exercises, participants performed a GXT on a treadmill using a +1% inclination. The initial speed was set at 8 km/h and was increased every minute by 1 km/h till exhaustion (Chrismas et al., 2017). During the late stages of the test, participants were cheered vigorously so that they made maximal effort. Measured HR_max_ was defined as the highest value attained during the test. HR was recorded continuously during the test by Team2 Pro (Polar Electro Oy, Kempele, Finland). Minute ventilation and VO_2_ were recorded by a gas analyzer (Fitmate Pro, Cosmed, Rome, Italy). Anaerobic threshold was identified from ventilatory threshold, i.e., the relationship between minute ventilation and oxygen uptake. Plateau of VO_2_ (primary criterion), blood lactate, age-predicted HR_max_ and RPE (secondary criteria) were used as criteria of VO_2_max (Howley et al., 1995). Desired RPE was ≥8 in the 0–10 of Borg scale (Borg, 1988). Blood samples were taken 5 min after termination of test, and lactate concentration was analyzed (Accutrend, Roche, Germany). Lactate concentration was employed as a criterion of VO_2_max achievement (accepted values > 9 mmol/L) (Todd et al., 2017). Predicted maximal heart rate was calculated using Tanaka's formula (Tanaka et al., 2001)—as Fox's formula might overestimate HRmax (Nikolaidis, 2015)—and was employed as a criterion of VO_2_max achievement (accepted values measured HR_max_ ≥ 95% of Tanaka-HR_max_).

### Statistical analyses

Statistical analyses were performed using IBM SPSS v.20.0 (SPSS, Chicago, IL, USA). Normality was examined using Kolmogorov-Smirnov test and visual inspection of normal Q-Q plots. Data were expressed as mean and standard deviation (SD). An independent *t*-test examined the sex differences in anthropometric and physiological characteristics. One-way repeated measures analysis of variance (ANOVA) and a subsequent Bonferroni *post-hoc* test (if there were differences among groups) were used to examine the differences between measured and predicted HR_max_. 95% confidence intervals (CI) of the mean differences were calculated. To interpret ES for statistical differences in the ANOVA, we used eta square classified as small (0.010 < η^2^ ≤ 0.059), medium (0.059 < η^2^ ≤ 0.138), and large (η^2^ > 0.138) (Cohen, 1988). Bland–Altman analysis was used to examine the accuracy and variability of prediction equations (Bland and Altman, 1986). Associations between measured HR_max_ and age were determined using Pearson's product moment correlation coefficient (*r*). Magnitude of correlation coefficients was considered as trivial if *r* ≤ 0.10, small if 0.10 ≤ *r* < 0.30, moderate if 0.30 ≤ *r* < 0.50, large if 0.50 ≤ *r* < 0.70, very large if 0.70 ≤ *r* < 0.90, nearly perfect if *r* ≥ 0.90, and perfect if *r* = 1.00 (Batterham and Hopkins, 2006). In addition, we used linear regression to model the prediction of HR_max_ from age in the total sample and in each sex. The linear regression was qualified for this analysis instead of non-linear regression as minimal differences among linear, quadratic, and polynomial equations have been shown (Ozemek et al., 2017). The level of significance was set at α = 0.05.

## Results

**Figure 1:**
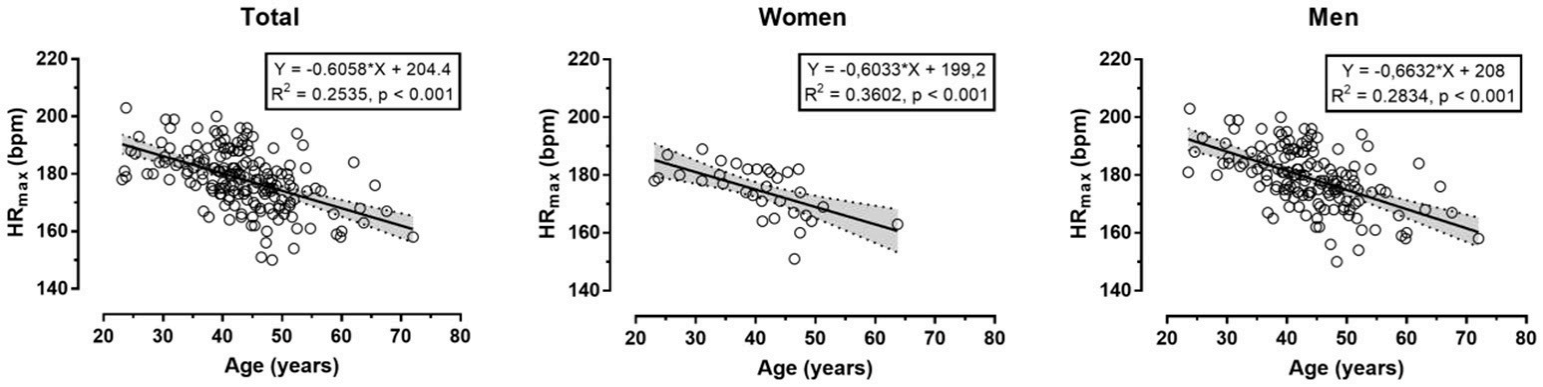
Relationship between measured maximal heart rate and age.

**Figure 2:**
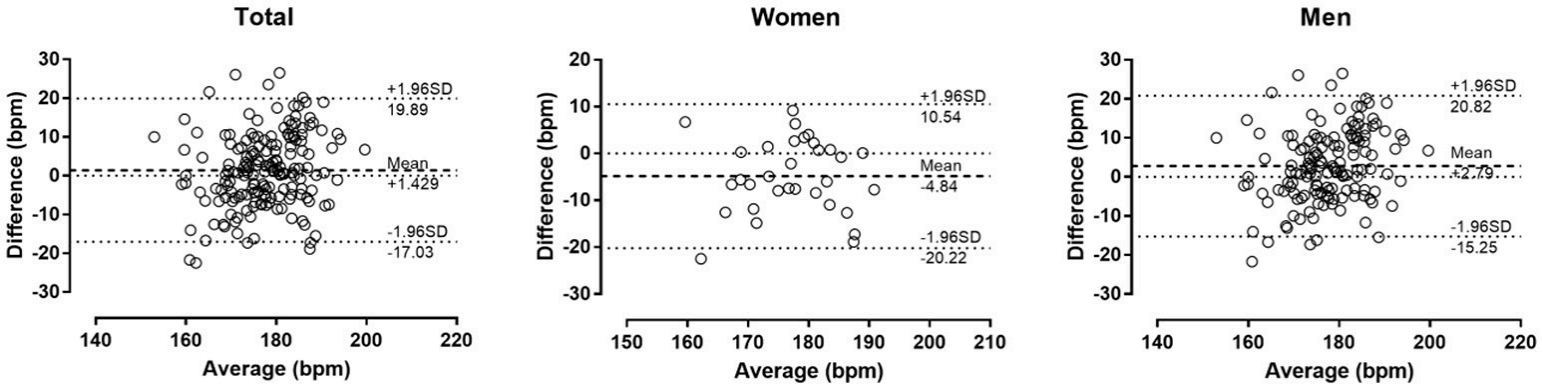
Bland-Altman plots of the measured maximal rate compared to the Fox's formula.

**Figure 3:**
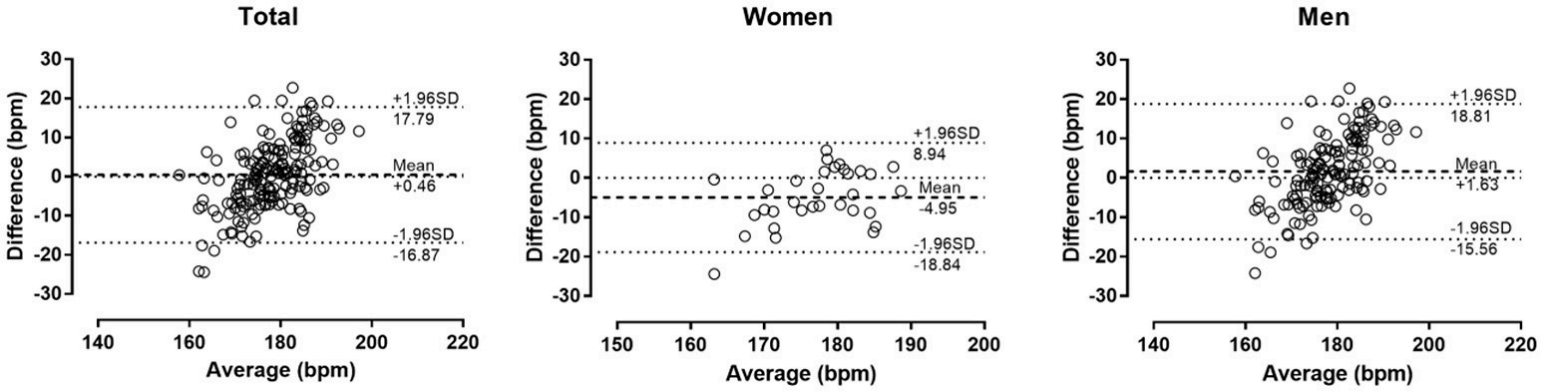
Bland-Altman plots of the measured maximal rate compared to Tanaka's formula.

The descriptive characteristics of participants are shown in Table 1. Measured HR_max_ correlated largely with age in the total sample (*r* = 0.50, *p* < 0.001), women (0.60, *p* < 0.001) and men (0.53, *p* < 0.001) (Figure 1). In the overall sample, no main effect of measured or predicted method on HR_max_ was observed (*p* = 0.093, η^2^ = 0.015); however, *post-hoc* comparison revealed trivially larger score in Tanaka-HR_max_ than in Fox-HR_max_ (mean difference +0.6 bpm, *d* = 0.12), whereas no difference was found between measured HR_max_ with Fox-HR_max_ (−1.0 bpm, *d* = −0.15) and Tanaka-HR_max_ (−1.7 bpm, *d* = −0.06). A moderate sex × assessment method interaction on HR_max_ was shown (*p* < 0.001, η^2^ = 0.087) with larger differences among methods in women than in men. In women, a large main effect of method on HR_max_ (*p* = 0.001, η^2^ = 0.294) was shown with measured HR_max_ lower than Fox-HR_max_ (−4.8 bpm, *d* = −0.56) and Tanaka-HR_max_ (−4.9 bpm, *d* = −0.66), and no difference between Fox-HR_max_ and Tanaka-HR_max_ (−0.1 bpm, *d* = −0.01). In men, a moderate effect of assessment method on HR_max_ was found (*p* = 0.001, η^2^ = 0.066) with measured HR_max_ higher than Fox-HR_max_ (+2.8 bpm, *d* = 0.30), Tanaka-HR_max_ higher than Fox-HR_max_ (+1.2 bpm, *d* = 0.17) and no difference between measured HR_max_ and Tanaka-HR_max_ (1.6 bpm, *d* = 0.19). The Bland-Altman plots are presented in Figure 2 (Fox-HR_max_) and Figure 3 (Tanaka-HR_max_). A visual inspection of these plots highlighted a trend that Tanaka-HR_max_ underestimated the low scores and overestimated the high scores in men.

**Table 1:** Descriptive characteristics of participants.

**Parameter**	**Total (*n* = 180)**	**Women (*n* = 32)**	**Men (*n* = 148)**
Age (years)	43.2 ± 8.5	40.3 ± 8.8	43.9 ± 8.3^*^
Height (cm)	173.7 ± 8.0	162.5 ± 6.6	176.1 ± 5.9^‡^
Body mass (kg)	73.6 ± 11.4	57.9 ± 7.4	77.0 ± 9.0^‡^
BMI (kg.m^−2^)	24.3 ± 2.7	21.9 ± 2.1	24.8 ± 2.5^‡^
BF (%)	18.0 ± 4.3	19.5 ± 4.7	17.7 ± 4.1^*^
VO_2max_ (mL/min/kg)	46.8 ± 8.9	37.5 ± 6.7	48.8 ± 8.1^‡^
Lactate (mmol/L)	10.8 ± 2.8	9.3 ± 2.6	11.2 ± 2.8^†^
RPE (a.u.)	8.8 ± 0.9	8.4 ± 1.0	8.8 ± 0.8^†^
HR_max_ (bpm)	178.2 ± 10.2	174.8 ± 8.8	178.9 ± 10.4^*^
Fox-HR_max_ (bpm)	176.8 ± 8.5	179.7 ± 8.8	176.1 ± 8.3^*^
Tanaka-HR_max_ (bpm)	177.7 ± 5.9	179.8 ± 6.1	177.3 ± 5.8^*^

BMI, body mass index; BF, body fat; VO_2max_, maximal oxygen uptake; RPE, rate of perceived exertion; a.u., arbitrary units; HR_max_, maximal heart rate; symbols

*, †, and ‡ *denote difference from women at p < 0.05, p < 0.01, and p < 0.001, respectively*.

## Discussion

The present study addressed the question whether the widely used age-based prediction equations of HR_max_, Fox's 220-age or Tanaka's 208-0.7 × age, are valid in recreation marathon runners since no study previously examined this topic. We hypothesized that these equations would overestimate HR_max_ in our sample, due to their expected lower HR_max_ compared to non-athletes (Zavorsky, 2000). The main findings were that (a) Fox-HR_max_ and Tanaka-HR_max_ overestimated HR_max_ by ~5 bpm in women, (b) Fox-HR_max_ underestimated HR_max_ by ~3 bpm in men, (c) Tanaka-HR_max_ was similar to measured-HR_max_ in men, and (d) the main effect of assessment methods on HR_max_ was larger in women than in men.

The overestimation of HR_max_ in women by age-based predicted equations was in agreement with previous findings (Esco et al., 2015). For instance, Fox and Tanaka formulas provided significantly higher estimates by 7–13 bpm compared with observed HR max in women collegiate athletes (Esco et al., 2015). Considering that the choice of assessment method had larger magnitude on women than in men, the overestimation of HR_max_ in women marathon runners is an issue that future research should address and develop sport-specific prediction equation.

The agreement between measured and Tanaka observed in men marathon runners was in line with previous research in young physically active men (Barboza et al., 2016), but not with a study on sedentary adults that showed that Fox and Tanaka-HR_max_ overestimated HR_max_ in sedentary adults by 2–4 bpm (Camarda et al., 2008). Camarda et al. (2008) found that Tanaka-HR_max_ overestimated HR_max_ only by 1 bpm in men. Tanaka-HR_max_ provided closer values to HR_max_ than Fox-HR_max_ in overweight adults (Franckowiak et al., 2011) and young physically active (Barboza et al., 2016). On the other hand, Fox-HR_max_ underestimated HR_max_ in older adults (Whaley et al., 1992). In male adults, Tanaka-HR_max_ underestimated HR_max_ by 5 bpm, while there was not any difference between Fox-HR_max_ and measured-HR_max_ (Nikolaidis, 2015). Differences between the findings of the present study and those of previous research should be attributed to the chronic physiological adaptations of recreational marathon runners to endurance training. Zavorsky (2000) highlighted that endurance training results in decrease of HR_max_ due to extrinsic/autonomic (e.g., plasma volume expansion) and intrinsic/non-autonomic factors (e.g., alteration of the electrophysiology of the sinoatrial node).

The moderate sex × assessment method interaction on HR_max_ indicated that sex should be considered in predicting HR_max_. Women marathon runners were younger by 3.6 years and had a 4.1 bpm lower measured-HR_max_ than men indicating a relatively lower HR_max_ if sexes were age matched. This observation was in agreement with a previous study showing difference in HR_max_ among sexes (Hakki et al., 1983).

The measured HR_max_ is in agreement with previous findings on age-matched humans (Arena et al., 2016); nonetheless the variation in our sample was smaller which should be attributed to the homogeneity of the sample. On the other hand, the slopes of the linear regressions suggested that HR_max_ decreases faster in men than in women, which was in disagreement with a previous study on healthy adults showing the opposite trend (Shargal et al., 2015). An explanation of this discrepancy might be the different samples' characteristic (age and sport).

A limitation of the present study was that it focused on the prediction of HR_max_ only from age excluding other parameters that might improve the accuracy of the prediction. For instance, Barboza et al. (2017) recommended an equation including age and HR at 150 W elicited during a GXT on a cycle ergometer in healthy young adult men. In another study, mode of exercise, fitness level, continent, and age were predictors of HR_max_ (Londeree and Moeschberger, 1982). Moreover, caution is needed to generalize the values obtained in the GXT in the laboratory to other settings, e.g., field testing, training and competition, as the latter might induce higher values (Coutinho et al., 2017). Nevertheless, strength of the present study was its novelty as it was the first to be contacted on recreational marathon runners. Considering the increasing number of those participating in marathon races, our findings are of great practical value for purposes of testing and training. Despite the different settings in laboratory and field, comparative studies observed no (Krautgasser et al., 2011; Alemdaroglu et al., 2012) or practically negligible difference (Meyer et al., 2003) in HR_max_ between these two conditions. Therefore, the findings of the present study could be applied in both laboratory and field settings, e.g., outdoors running training sessions. In addition, exercise physiologists performing exercise testing should benefit from such knowledge in order to evaluate correctly HR as criterion of achievement of VO_2max_.

## Conclusions

Based on the present findings we recommend the further use of Tanaka's formula in men recreational marathon runners with similar training characteristics as those of the participants in the present study. In addition, exercise physiologists and sport scientists should consider the observed differences among various assessment methods when performing exercise testing or prescribing training program relying on HR.

## Author contributions

PN performed the laboratory analyses, the statistical analyses and drafted the manuscript; TR and BK helped in drafting the manuscript.

### Conflict of interest statement

BK was employed by Medbase St. Gallen Am Vadianplatz. The other authors declare that the research was conducted in the absence of any commercial or financial relationships that could be construed as a potential conflict of interest.
